# Commentary: Neutrophil-Related Ratios Predict the 90-Day Outcome in Acute Ischemic Stroke Patients After Intravenous Thrombolysis

**DOI:** 10.3389/fphys.2022.936860

**Published:** 2022-06-15

**Authors:** Youwei Lin MM, Qianghong Xu MM

**Affiliations:** ^1^ Yuhuan Second People’s Hospital, Taizhou, China; ^2^ Department of Intensive Care, Zhejiang Hospital, Hangzhou, China

**Keywords:** granulocyte, multicollinearity, acute ischemic stroke, poor outcome, events per variable

## Introduction

In a recent study (Gao et al., 2021), investigated the neutrophil-related ratios in patients with acute ischemic stroke after thrombosis. After adjusting for confounders, they reported several granulocyte parameters were independently associated with 90-day outcomes. This study adds important information to the current evidence. However, several points may need to be noted when interpreting these findings.

## Multicollinearity

In the multivariable logistic regression model, eight granulocyte parameters, including neutrophil ratio, neutrophil count, lymphocyte count, eosinophil count, monocyte count, neutrophil to lymphocyte ratio (NLR), neutrophil to eosinophil ratio (NER), and monocyte to neutrophil ratio (MNR), were including in the same model. In model 2, neutrophil ratio, neutrophil count, NLR, NER, and MNR were significantly associated with poor neurological outcome at 3-month (mRS 3-6). However, we should note that these granulocyte parameters may have a close relationship with each other. Simply including these parameters in the same model may increase the risk of multicollinearity (Vatcheva et al., 2016; Kim, 2019), which may lead to a biased estimation. Besides, when the results of the multivariate analyses change greatly from that of the univariate analyses (Zhang and Zhu, 2022), we suggest that multicollinearity, modulating effect, and mediation effect should be considered in addition to interacting effect.

## Events per Variable

In addition, in the current study, 283 patients with AIS were included, 87 of whom had positive events (poor neurological outcome at 3 months). A total of seventeen variables were included in the multivariate logistic model, and the number of events per variable (EPV) is 87/17=5.1. However, according to Peduzzi et al.‘s finding (Peduzzi et al., 1996), the EPV less than 10 may also increase the bias risk of the regression coefficients.

Finally, this study provided important information for acute ischemic stroke, and their work is very much appreciated.
